# ‘We've Taken on a More Advanced Clinical Role’: A Multimethod Study of Community Nurses' Extended Roles in Palliative Care

**DOI:** 10.1111/jan.70019

**Published:** 2025-06-17

**Authors:** Ben Bowers, Kristian Pollock, Simon Etkind, Alison Leary, Stephen Barclay, Louisa Polak

**Affiliations:** ^1^ Department of Public Health and Primary Care University of Cambridge Cambridge UK; ^2^ School of Health Sciences University of Nottingham Nottingham UK; ^3^ Institute of Health and Social Care London South Bank University London UK

**Keywords:** community care, district nursing, multiprofessional care, nurse roles, palliative care, practice‐based learning, professional development

## Abstract

**Aim:**

To explore community nurses' experiences of changes to their roles in palliative and end‐of‐life care.

**Design:**

An e‐survey was followed by focus groups.

**Methods:**

Fifty‐one community nurses with recent experience of delivering end‐of‐life care in the United Kingdom completed a survey about changes to their roles. A purposive sample of 35 respondents participated in focus groups exploring these changes in more depth; thematic analysis was used with constant comparison.

**Results:**

As well as two new roles—prescribing and verifying death—many participants talked about a broader expansion of their role, increasing their leadership in making complex end‐of‐life care decisions with patients and families. Most nurses expressed pride in their new knowledge and skills, and satisfaction with the care they were providing. Yet many also expressed distress that heavy workloads impaired their capacity both to provide good clinical care and to train junior colleagues. The importance of General Practitioner support with complex cases was often highlighted, but accessing such support was sometimes difficult.

**Conclusion:**

While welcoming the opportunity to extend their palliative care roles, many participants indicated experiencing moral distress.

**Implications for the Profession and/or Patient Care:**

Excessive workloads and patchy medical support threaten the retention of the experienced nurses upon whom community palliative care depends.

**Impact:**

Our findings suggest that new and extended palliative care roles are viewed positively by nurses. To be sustainable, these changes require better workload management and consistent medical back‐up.

**Reporting:**

We adhered to relevant EQUATOR guidelines, using the SRQR checklist.

**Patient or Public Contribution:**

Our Public and Clinician Advisory Group helped shape questions and commented on findings.

**Summary**

- Relevance to the wider global clinical community
  ○ Demand for community palliative care is growing in many countries.
  ○ Nurses' central role in providing this care is widely recognised, as is the importance of interdisciplinary collaboration and support.

## 1 Introduction

### 1.1 Background

Community nurses have always worked very closely with patients and their families at the end of their life. Two big changes have affected this work in recent years; both were accelerated by the 2020–2021 COVID‐19 pandemic (Mitchell, Barclay, et al. 2022) and have continued evolving since. First, more people are dying from chronic illnesses worldwide (Sleeman et al. 2019), and an increasing proportion of these deaths are taking place at home so there is more community end‐of‐life care to provide (Sleeman et al. 2019; Bone et al. 2018; Office for National Statistics 2024; Sørstrøm et al. 2025; Naruse et al. 2017; Modin et al. 2010). Community nurses' palliative (or ‘end‐of‐life’) caseloads have consequently increased, alongside the complexity of their patients' needs (Marie Curie, 2021, 2024) Second, in the United Kingdom, changes in the way general practitioners (GPs) and palliative specialist teams work have reduced the amount of face‐to‐face contact these clinicians offer (Williams et al. 2023), with remote consultations replacing some face‐to‐face ones and GPs making fewer home visits (Mitchell et al. 2020, 2021). This second change has placed community nurses, some with the title of district nurse, even more centrally than before within the complex patchwork of community palliative care provision. Nurses now describe themselves (Robinson et al. 2023) as taking the lead in the many aspects of this care that require face‐to‐face interactions, for example facilitating sensitive conversations and assessing a distressed patient (Griffiths et al. 2015); we use the adjective ‘extended’ to identify these more in‐depth roles. These were activities that many nurses felt ill‐prepared for before the pandemic, having previously looked to GPs and palliative specialist teams for leadership and input (Walshe 2020; Dees et al. 2018).

### 1.2 The Study

This study explores community nurses' views and experiences of their new or extended roles in palliative and end‐of‐life care. We build on research conducted during the pandemic into clinicians' experiences of providing community palliative care during that crisis (Antunes et al. 2022; Mitchell, Harrison, et al. 2022; Turner et al. 2023). What is new here is our in‐depth focus on community nurses' perceptions of their roles, and our timing: we conducted this study after the pandemic had waned and services had begun establishing their ‘new normal’. These services rely on an adequate workforce, and retaining experienced community nurses is especially crucial if they are to continue fulfilling the roles assigned to them. Yet recruiting and retaining community nurses can be difficult, as their falling numbers in the United Kingdom (NHS England, n.d.) show. This poses a challenge to the sustainable provision of community palliative and end‐of‐life care. To address that challenge, healthcare commissioners and providers need the nuanced understanding of nurses' recent experiences that this study aims to offer.

## 2 Methods

### 2.1 Design

This was a multimethod study. An e‐survey generated a descriptive overview of some specific areas of change in participants' palliative and end‐of‐life care roles since the beginning of the pandemic. Survey results provided a robust starting point for our subsequent in‐depth qualitative exploration of community nurses' perceptions of their new or extended roles. This exploration used focus groups, adopting a social constructionist approach (Berger and Luckmann 1966). The survey facilitated recruitment to focus groups. Data from these two arms of the study were analysed separately.

### 2.2 Ethical Approval

Ethical approval [PRE.2022.098] was obtained from the University of Cambridge Psychology Research Ethics Committee on 20th January 2023.

### 2.3 Recruitment Procedure and Inclusion/Exclusion Criteria

Eligible participants were registered nurses working in community nursing teams in the United Kingdom who had cared for adult patients (aged 18+ years) at their end‐of‐life in the community within the last 3 months. We did not recruit nurses working in specialist palliative care teams.

To identify potential participants, brief information on the study and an expression of interest e‐response form were shared through UK‐wide community nursing professional networks and via X (formally Twitter) and Facebook. Recipients were encouraged to forward the information to colleagues, seeking a snowball sample of community nurses.

Fifty‐one respondents completed the e‐survey, of whom 35 later participated in focus groups. The survey enabled purposive sampling of focus group participants, with the aim of interviewing interested nurses from a range of geographical areas, and with a range of levels both of professional experience and of pay grade or ‘Band’.

### 2.4 Data Collection

Data were collected between February and April 2023, about a year after UK lockdowns ended. Participants provided written informed consent. The questionnaire‐based e‐survey (Appendix S2) explored participants' characteristics, end‐of‐life care experience and perceived changes in their clinical roles using a 5‐point Likert scale. These questions drew on an earlier survey study (Mitchell et al. 2021) and were shaped with Public and Clinician Advisory Group members.

Ten focus groups were conducted with 35 e‐survey respondents, chosen purposively to give a good spread both geographically and as regards banding. These were facilitated by a postdoctoral research assistant and audio‐recorded. A semi‐structured interview topic guide (Appendix S3) was used to explore participants' experiences of taking on new or extended clinical roles since the start of the COVID‐19 pandemic. All interview transcripts were transcribed verbatim by a professional transcriber, anonymised and checked for accuracy.

### 2.5 Analysis

E‐survey responses were analysed descriptively for frequency and mean by using SPSS version 24. By establishing that most participants presented themselves as having taken on new or extended palliative and end‐of‐life care roles since 2019, this preliminary analysis reinforced the validity of our subsequent qualitative exploration of nurses' perceptions of these changes.

Qualitative data were analysed inductively using thematic analysis (Braun and Clarke 2019) with constant comparison and careful attention to deviant cases (Green and Thorogood 2018). Initial close coding by L.P. and B.B. allowed us to produce a detailed description of salient themes within the data. In subsequent iterative cycles of analysis, L.P., B.B. and K.P. used constant comparison to draw out differences and contrasts within these data, constructing a theoretically informed account that makes sense of what is going on here.

Later in the process, the key findings were discussed with our research group, an interdisciplinary team of people interested in community palliative and end‐of‐life care. Professional backgrounds within the group include nursing, general practice, geriatrics, public health, sociology and specialist palliative care. Group members were particularly interested in the challenge of recruiting participants who were not particularly enthusiastic about palliative care; we have reflected on this as a limitation of the study. In addition, several clinicians explicitly endorsed our foregrounding of moral distress and perceived overwork within the data and spoke of potential burnout as a probable threat to retention.

### 2.6 Rigour and Reflexivity

To aid rigour and reflexivity, L.P. (qualitative researcher and retired GP), K.P. (medical sociologist) and B.B. (clinical academic community nurse) worked closely together to develop the coding frame and analysis of the data. L.P.'s and B.B.'s respective clinical backgrounds provided helpful perspectives on the data, while K.P. used her extensive experience as a social scientist to ensure that these perspectives were used with appropriate reflexivity. Early analytical insights and results were discussed with co‐authors and the study's Public and Clinician Advisory Group (two family carers and five community nurses from across the United Kingdom) to gain their reflections and consider the implications for practice.

## 3 Findings

After providing some information about our participants, we shall present the results of the survey, followed by findings from the qualitative arm of the study.

### 3.1 Participant Characteristics

Appendix S1 presents relevant characteristics of participants both in the survey and in the focus groups. To describe participants' level of seniority we refer to their ‘band’: Banding is a pay structure and grading system used in the UK's National Health Service (NHS) to categorise nurses based on their responsibilities and the skills their role requires. ‘Band 5’ includes newly qualified nurses; higher bands indicate higher pay and increasing levels of responsibility, including managing teams.

Focus groups varied in size between two and eight participants. In total, 11 band‐5 nurses took part, 11 band‐6 and 13 band‐7 or ‐8 nurses. We arranged groups with a broad geographical spread (see map) to elucidate variations between areas. We also aimed for groups with a narrow spread as regards banding, in case junior participants felt awkward about speaking in front of more senior peers. However, it was quickly apparent that there was little correlation between banding and years of community nursing experience; this may explain why our data did not surface any salient differences between participants with different banding levels, except that the work of training others was mentioned only by nurses with banding levels above 5.

### 3.2 E‐Survey Results

As Table 1 shows, participants reported having a variety of new roles or extensions of their old roles in palliative and end‐of‐life care following the pandemic. Most participants reported having advance care planning conversations with patients and families more often than prior to the pandemic, and asking more about preferences regarding cardiopulmonary resuscitation, although only a minority said they were completing more forms to record these plans and preferences. The majority of respondents also reported making more clinical decisions about end‐of‐life symptom management, and many more said they were undertaking verification of death. A minority of respondents were qualified prescribers. There was considerable diversity in perceptions of changes in collaborative working across inter‐professional boundaries.

**TABLE 1:** Self‐reported changes in community nurses clinical roles since the onset of the 2020–2021 COVID‐19 pandemic.

Questions	Responses (*N* = 51)	Percentage
**Having advance care planning conversations with patients and families**		
Doing less	1	2
Stayed the same	11	22
Doing more/a lot more	39	76
**Asking patients and families about their preferences for cardiopulmonary resuscitation**		
Stayed the same	21	41
Doing more/a lot more	30	59
**Completing the ReSPECT form/do not attempt cardiopulmonary resuscitation (DNACPR)**		
Doing a lot less	3	6
Stayed the same	28	55
Doing more/a lot more	20	39
**Making clinical decisions about the prescribing of anticipatory medications (injectable medications)**		
Doing less	1	2
Stayed the same	17	33
Doing more/a lot more	33	65
**Prescribing injectable anticipatory medications, yourself**		
Stayed the same	6	12
Doing more/a lot more	8	16
N/A—not a prescriber	37	72
**Initiating/requesting the remote prescribing of anticipatory medications**		
Stayed the same	10	20
Doing more/a lot more	41	80
**Carrying out joint virtual consultations with general practitioners: where you are with the patient and family and the general practitioner joins you by phone or video**		
Doing less/a lot less	15	29
Stayed the same	20	39
Doing more/a lot more	16	31
**Collaborative working with general practitioners**		
Doing less/a lot less	21	41
Stayed the same	9	18
Doing more/a lot more	21	41
**Collaborative working with specialist palliative care teams**		
Doing less/a lot less	12	23
Stayed the same	12	23
Doing more/a lot more	27	53
**Meeting with the multidisciplinary team, in‐person or remotely, to discuss patients identified as being in their last year of life**		
Doing less/a lot less	13	25
Stayed the same	22	43
Doing more/a lot more	16	31
**Making clinical decisions about end‐of‐life symptom management**		
Stayed the same	12	23
Doing more/a lot more	39	77
**Administering (giving) injectable medications to manage symptoms in the last days of life**		
Doing less	3	6
Stayed the same	22	43
Doing more/a lot more	26	51
**Verifying death**		
Doing less/a lot less	2	4
Stayed the same	8	16
Doing more/a lot more	35	69
N/A—not an option in my area	6	12
**Providing bereavement support for family members and carers**		
Doing less/a lot less	7	14
Stayed the same	18	35
Doing more/a lot more	26	51

Abbreviation: N/A, not applicable.

### 3.3 Qualitative Findings, From Focus Groups

The community nurses who took part in this study identified three salient changes to their roles in palliative and end‐of‐life care since the start of the COVID‐19 pandemic in 2020. They described two new roles that were clearly defined and demarcated: verifying death and prescribing medication. Participants also spoke of an important extension to their pre‐existing role within the healthcare team providing palliative and end‐of‐life care in the community, describing a shift through which they had taken on increased leadership of care coordination and clinical decision‐making. We shall first present data about these three new or extended roles and then examine participants' feelings about the changes, which included both pride and dissatisfaction.

#### 3.3.1 New or Extended Roles

Several participants mentioned verification of death as a new role they had taken on during the pandemic and continued since, as the following example shows.

The verification wasn't really something we'd done before and actually what we've found now is that has carried on and we do the majority of the verification of expected deaths. FG1 [P04]

The other clearly demarcated new role that some Band 6 and 7 nurses had taken on was prescribing of end‐of‐life symptom control medications:

I've had the opportunity to do my V300 [prescribing qualification] since the pandemic and what we do now […] there's […] generally always a prescriber on for End‐of‐Life patients on a weekend, and we didn't have any of that before Covid. FG2 [P08]

In addition, most participants talked about an important but less clear‐cut extension of their previous role; they described taking on more leadership of decision‐making in complex situations. This role involved facilitating in‐depth discussions and shared decision‐making with patients and their families, as well as liaising with other care providers to coordinate care. For participants who were prescribers, this complex deciding and coordinating work was closely entangled with the work of prescribing palliative medication. Where the patient was currently comfortable and stable, for example, proactive discussions and decisions informed the prescription of injectable anticipatory medications for future symptom control close to the end of life. Encounters with an acutely distressed patient involved the prescriber assessing a complex situation and making a plan of action, as well as making decisions about which drugs to administer, when and in what dosage.

Proactive discussions were often mentioned in accounts of helping patients and their informal carers complete forms that record their preferences regarding end‐of‐life care. Many identified this as an area where demand had increased.

There was a lot more requests for ReSPECT [personal emergency care plan summary] … so a lot more discussions around the ReSPECT wishes and doing Do Not Resuscitate forms FG8 [P41]

A related area of proactive discussion, advance care planning, overlaps with these emergency plans and forms, but covers a wider range of topics. Although some participants mentioned this as something that was already part of their remit before the pandemic, others identified it as another key area where their role had extended. As well as saying that they were now ‘doing a lot more advance care planning’ FG4 [P122], several indicated that they were more deeply involved in the delicate and complex task of establishing the patient's preferences before recording these, a task that GPs and palliative specialists typically used to do before the pandemic.

Whereas in the past we might have had a small conversation and then passed that over to Macmillan [specialist palliative care nurses], I think now we are more involved in that, that side of things. FG3 [P13]

Liaising with other helping agencies is another role that has been extended and deepened.

I get a lot more requests to go and do the complex management of patients, the having those anticipatory conversations, ReSPECT conversations, looking at their social care needs FG1 [P07]

Several participants related this change to a shifting of roles and responsibilities from GPs and other clinicians.

We do all the discussions with patients, referring to Macmillan, it's a lot more in‐depth for us than it was before. The GPs seem more remote now and we seem very heavily involved […] it's totally switched round really FG4 [P18]

In addition to this deeper involvement in proactive ‘complex management’ in relatively stable situations, many participants described an extension of their assessment and decision‐making roles in response to an urgent problem. Here again, several identified this as taking on work formerly done by other clinicians.

We've taken on a more advanced clinical role, a lot more clinical assessment that would have been undertaken by a GP or a palliative nurse specialist and we're doing a lot more of that. FG10 [P45]

Together, these new and extended roles are part of a broad shift of responsibility portrayed by participants as placing them in the driving seat of palliative and end‐of‐life care. The rewards and burdens associated with this shift are the focus of our next two sections.

#### 3.3.2 Pride and Satisfaction About New Roles

Many participants spoke very positively about their experiences of providing community end‐of‐life care in general, and about their new or extended roles in particular.

I think in some ways it [*shift of DNACPR conversations to nurses*] was quite positive because it did really play to kind of the skillset that community nurse and district nurses have, you know that's one of our specialist areas that we do really well in FG1 [P02]

Some identified an extension to their role as a benefit of the sudden task‐shifting prompted by the COVID‐19 pandemic.

It's allowed me to go on and sort of demonstrate that yes I wanted to do my non‐medical prescribing […] and I feel really lucky I've been supported to do that and it makes a massive difference in this area FG2 [P08]

Elsewhere in the data, however, these sudden changes and the ways in which they came about were spoken of with a marked resentment that seemed to colour participants' perceptions of their colleagues both within and outside the nursing profession. Several presented such negative perceptions alongside their satisfaction about extended roles, qualifying their pride in having done a good job both during the pandemic—‘we were fantastic, but it appears to be the other services that really let us down’ [FG1]—and since it ended:

We've become now more skilled because of the pandemic, which is brilliant for us and my confidence has grown but […] GPs now seem more willing to leave you to it FG4 [P18]

In this context, being ‘left to it’ was clearly framed as unwelcome—an implicit criticism of GPs, rather than an indication of their respect for nurses' competence. To understand nurses' perceptions of their extended roles, then, it is essential to think about how their positive feelings articulate with the concern and dissatisfaction they express.

#### 3.3.3 Dissatisfactions and Resentments

Participants expressed negative feelings about several aspects of their new roles. These feelings were about two broad issues, with some overlap between them: dissatisfaction about having too much work and unhappiness at having too little support when making complex clinical decisions.

##### 3.3.3.1 Too Much Work—Moral Distress

Participants were unanimous that their workload was higher now than it used to be. Many said they felt it placed a lot of pressure on them; several indicated that this pressure caused them distress by constraining their ability to do what they felt needed doing. As well as patient care, this constraint was felt in relation to experiential learning: senior nurses described struggling to mentor junior colleagues, and some more junior participants regretted the paucity of opportunities for shadowing experienced peers and debriefing.

Many blamed new and extended roles for these experiences of overwork.

There's […] no extra time given to do these extra roles, and that's you know, where the negative for me comes in, […] it's that constant pressure […] And then you feel well have you given everything that you could to that person, and that family? FG3 [P13]

A sense of unease is surfaced by this last excerpt; the closing question is clearly rhetorical, indicating that the speaker feels ‘constant pressure’ has constrained their ability to provide the care they feel they ought, a standard definition of moral distress.

As well as in relation to their clinical work, further moral distress was visible in senior nurses' accounts of wanting to support and train less experienced colleagues. Here, as in many accounts, the current unsatisfactory state of affairs was contrasted with an unspecified time ‘previously’ when junior colleagues were given an opportunity to learn through observation and supervision:

As [Band] 6s and 7s [we…] go and do those more advanced things which previously we would've taken the Band 5s with us to do FG1 [P01]

In contrast, now:

Seeing it done, that's exactly what's missing, you need that experience of going out with other people to see how you would actually go forward in doing it, and that's what our staff aren't getting. FG7 [P39]

Several nurses emphasised that this experiential learning was indispensable: the confidence and competence to lead ‘difficult conversations’ is something that ‘just comes with experience’ and with ‘observing them [senior colleagues] having difficult conversations and them giving me advice’ FG10 [P44].

Some senior participants described managing to continue this training work, sometimes by prioritising it over clinical care:

We do make sure we schedule regular supervision, which is incredibly important, and like live supervision as well where we have a senior nurse going out with the more junior staff […] No matter how busy we are we will always have a clinical update, like a handover so people can debrief and talk about their visits and raise any concerns. So that is prioritised and we do try and protect supervision time, so there might be patients that are deferred FG8 [P41]

Others, too, said that they managed intolerable pressure by refusing work that they could not fit into their working day:

We have caseloads, in the hours that I work is supposed to be forty to fifty [patients] and it hasn't been under sixty‐five for the last three years, so yeah, you do have to say ‘well actually I can't do that’ FG8 [P40]

Keeping the workload manageable in this way, however, may be in tension with the perceived imperatives both to support their team—

I will often end up, when I'm not even working, supporting staff when I'm not on duty and not paid. So, I'm very often working seven days a week probably 8.00 in the morning till 8.00 in the evening FG1 [P03]

—and to do ‘the best that [one] can for those patients’—

I work lots on my days off, and […] I always find myself in the evenings logging back on, just to double‐check […] So but then I have job satisfaction that I know I've done the best that I can for those patients, and I, that's the only way I can sleep at night. FG7 [P37]

These participants, then, regularly work in their own time in order to feel satisfied with the quality of their work.

Feelings of moral distress and excessive workloads are compounded by three perceived issues. First, participants felt that community nurses were not accorded enough acknowledgement and respect, either by colleagues or the public.

[speaker 1] ‘We're the forgotten service aren't we […] we're not recognised for actually the complexity of the roles that we're undertaking’

[speaker 2] ‘I don't think people know what we do, and I think they get us confused with carers’ FG3 [PP12&10]

Second, they felt that their pay grade was not commensurate with their responsibilities and not as high as that of other nurses with equivalent responsibilities.

I was a Band 6 in the hospital and then I came out to community and dropped, and you would have to go on the district nursing course to be a qualified district nurse, and for the same qualification, the health visitor gets a 7, a midwife gets a 7, but we're Band 6 FG9 [P42]

Third, participants felt that they were insufficiently consulted about the big changes that had occurred to their roles.

It's just put on us, ‘Can you do this?’, ‘This is the training you need to attend.’ There's no real consultation FG3 [P10]

In contrast with this comment about the present, however, a lack of consultation was felt to have been acceptable during the pandemic, when other healthcare professionals also had to put up with being given new roles without any consultation or choice.

We didn't really get any choice at that time *[laughs]*, ‘You need to come and do this training.’ I suppose it was an unknown wasn't it the pandemic, […] I know a lot of other staff who were redeployed into areas they'd never worked in FG2 [P08]

From these community nurses' perspectives, however, the trouble was the tacit assumption that they would continue fulfilling new roles they took on during the pandemic. Although many explicitly said they were happy with these roles, the comments quoted above show that there was also a widespread unhappiness and resentment about being taken for granted.

##### 3.3.3.2 Too Little Support With Complex Clinical Decisions

Compounding these dissatisfactions about the quantity of palliative care work expected of community nurses, we also elicited several accounts of unease about the kind of work they were being asked to do, and in particular about tasks where they used to rely on advice and support from GPs and specialist nurses. This unease was not unanimous in our data—it varied between different tasks, as well as between individuals. Tasks concerning patients' preferences for future care were not a visible focus of anxiety here, although several nurses spoke of ‘difficult conversations’ that they now initiate and lead (having not done so in the past) and mentioned the emotional toll of this work. In contrast, many nurses did indicate concern about taking a lead role in assessing and managing the situation when a patient develops distressing symptoms.

It feels like there's an overreliance on the community nurses especially to diagnose the problem and then the GPs will just react to whatever we're telling them without actually seeing the patient face‐to‐face. FG8 [P41]

As in this excerpt, concern about ‘overreliance’ is linked to the perceived inadequacy of GPs' support in these complex situations. The next speaker frames this point more positively:

I think that's such an important part of nursing at home is that we need the GP back‐up. FG1 [P01]

Participants spoke about two barriers to getting the clinical support they wanted: there were practical difficulties in accessing GPs, and even when they could be accessed, many GPs were perceived to lack adequate palliative care expertise.

Ease of accessing GPs was reported to vary widely from one general practice to another, sometimes within the same area. Good access included opportunities for face‐to‐face conversations and easy contact by phone, both preferably with a known GP. Conversely, never meeting in person and not building a relationship with the GP over time was problematic. Many practices encouraged electronic messaging as the main mode of communication, but most nurses work with several different practices, each with its own electronic system to navigate.

We used to have a brilliant relationship with our GPs, we used to work alongside them, we could go see them any time we wanted, you could stand outside the door in the practice, but now we've changed to geographical working, you cover three or four different surgeries […] I don't know any of the GPs now, I very rarely speak to them, each one has a different way of communicating, emails, tasks, different systems to write on, the relationships are so fractured FG4 [P18]

Lack of easy phone access was often identified as a source of irritation and wasted time; several nurses described having to join the same phone queue as patients and then leave messages with GPs' receptionists or administrators.

I feel like they're putting a lot more on their receptionists as well, so trying to get through, oh, past the receptionists, you know, when you're needing just in case medication [injectable anticipatory medication], or you need to review the dose […] it's so frustrating because you just think if you just put me through to the GP it would take me two minutes to explain it FG7 [P38]

In addition to these organisational issues, many participants expressed concern and dissatisfaction about the variable quality of GP advice, some describing it as often inadequate. A common story was about GPs asking the community nurses for advice about prescribing palliative medications:

The GPs look to us a lot for advice […] and a lot for guidance on what dosages to prescribe. […] A lot of the GPs are very unsure if they've not had much exposure to palliative care throughout their training. We've got a lot of very young GPs as well. FG10 [P45]

The lack of established inter‐professional working relationships, particularly with GPs, was stated to be particularly problematic in relation to out‐of‐hours care.

We would have had a very good relationship with some of our elderly GPs who have since retired and locums have stepped in there now […] [with] absolutely no knowledge of the paperwork or the documentation, the policies and the procedures […] So when you're on at the weekend you literally have a white‐knuckle ride because you do not know who you are getting […] you've no idea what their competencies are or what their knowledge or anything is. FG4 [P16]

In summary, the feeling that ‘we need the GP back‐up’ was widely visible in our data; by implication, the extension of nurses' roles to encompass autonomous leadership of care was presented as unwelcome, particularly in the context of assessing and managing an acutely distressed patient. Satisfaction with the back‐up currently available varied widely, with some participants describing good working relationships with known and trusted GPs. Many others, however, indicated marked dissatisfaction that centred around fractured relationships with their GP colleagues and practical barriers to communicating with them, as well as a lack of confidence in GPs' knowledge and competence.

## 4 Discussion

This study provides an in‐depth account of the evolving roles of community nurses delivering palliative and end‐of‐life care. New and extended roles have developed rapidly, particularly since the COVID‐19 pandemic. These roles were largely viewed positively by participants, but many also expressed unhappiness about increased workload and patchy clinical support.

Some of these findings are unsurprising, although nonetheless important. Perceived overwork, for instance, compounded by moral distress concerning patient care, has been widely reported amongst healthcare professionals since the COVID‐19 pandemic (MDDUS 2023); community nurses' wish to be involved in planning and refining changes to their roles, and remunerated appropriately for taking on additional responsibilities, is not surprising either. Several other findings, however, are more unexpected, or add nuance to what is already known. In addition to the strong positive tone of many participants' comments about their new or extended roles in community end‐of‐life care, two further points stand out: the importance community nurses ascribe to facilitating experiential learning within their teams; and the unhappiness and anxiety visible in many participants' accounts of getting less ‘GP back‐up’ than they feel is needed.

### 4.1 Supporting Experiential Learning

As well as the expected accounts of frustration at not having enough time to fulfil their (mainly welcomed) new clinical roles ‘properly’, several of our more senior participants also indicated moral distress regarding their long‐established role in educating and mentoring junior colleagues. They emphasised the importance of finding enough time to enable reflective experiential learning through joint visits and careful debriefing, both for themselves and more junior team members. Although our direct questions about education were often answered in terms of being encouraged to attend courses, participants emphasised that having ‘difficult conversations’, for example, cannot be learned from a book. These indications accord with Schon's (1987) seminal work on developing expertise through reflection‐in‐action while working in ‘the swampy lowlands’ of everyday professional practice.

In the specific context of education for palliative and end‐of‐life care, our findings echo Danielsen et al.'s (2018) point that regular supportive debriefing and mentoring were seen as essential by clinicians providing this care; likewise, Selman et al. (2017) found GPs preferred supported experiential learning about palliative care to didactic methods underpinned by behavioural assessments. To train and retain a new generation of community nurses able to provide effective care, commissioners and service managers need to ensure that their systems facilitate experiential learning, for instance by allocating time for junior nurses to shadow more experienced colleagues, a core element of their continuing professional development. Providing this additional time is likely to cost more than simply offering ‘targeted interventions’ intended to promote adherence to improved ‘unified guidance’, as recommended elsewhere (Turner et al. 2023), but we argue that the experiential approach is likely to be more effective in developing and supporting autonomous practitioners.

Our findings also suggest that supported experiential learning needs to be underpinned by a change in the approach perceived to be used by nurses' regulators, shifting away from punitive responses where decisions are judged to have been wrong. Clinicians' anxiety about such decisions must be understood in the context of a series of high‐profile cases in the United Kingdom of overtreatment with opioids (Pocock et al. 2018); the caution this engenders may promote under‐treatment (Heneka et al. 2018), leading some community nurses to frame end‐of‐life symptom control medication decisions as balancing two risks, over‐ and under‐treatment (Wilson et al. 2015).

### 4.2 Strengthening ‘GP Back‐Up’

The other way to address nurses' concerns about taking on leadership of these challenging assessments and decisions is one many participants suggested: rebuilding strong working relationships with local GPs. Previous studies (Staats et al. 2018; Bowers et al. 2020; Pornrattanakavee et al. 2022) have highlighted the importance of such relationships, and the systems that support them, in enabling safe and effective collaboration between GPs and community nurses. Robust interdisciplinary collaboration is widely recognised as a key component of palliative care in many countries and settings, affecting patient outcomes such as place of death (Naruse et al. 2017) and symptom control (Pornrattanakavee et al. 2022) as well as nurses' morale (Pence 2008).

Patchy access to GP support is only half the problem, according to our participants; the other half is the patchy perceived quality of such support. Nurses' lack of confidence in GP expertise regarding end‐of‐life care, and their particular concerns about the quality of GP support available out‐of‐hours, are mirrored by a reported (Wyatt et al. 2022; Jones et al. 2023) lack of confidence amongst GPs themselves. The dual challenge for policy‐makers and commissioners is to rebuild GPs' confidence and expertise, and to foster the rebuilding of effective relationships within the multidisciplinary teams that provide community palliative and end‐of‐life care.

## 5 Strengths and Limitations

Strengths of our study include the recruitment strategy and timing, and an analytic approach that used comparisons both within the data and with what is already known.

The breadth of recruitment enabled us to elicit contributions from nurses working in a wide range of geographical areas, including both rural and urban areas in England and Scotland. The study's timing enabled us to move on from previous studies' focus on ‘pandemic‐related changes’, instead building a picture of nurses' perceptions of their current roles and their ideas about how things might be improved in future.

Iterative cycles of analysis using constant comparison allowed us to produce an in‐depth, nuanced description of what was going on in the data. By identifying many ways that these data accord with the findings of other studies conducted in different countries, we situate our account as a contribution to the growing international literature on community nurses' core roles within palliative care.

As regards limitations, we acknowledge that invitations to participate in a palliative care study are likely to be accepted mainly by enthusiasts for this area of nursing practice; it is possible that the findings would be less positive if participants were included who found palliative and end‐of‐life care unappealing or excessively challenging. To address this potential selection bias, a future study might invite nurses to talk more generally about their work, and then include a question about palliative care. We are also mindful of the old caveat about what can be learned from interviews: be wary of drawing inferences about what people do from what they say they do. Nonetheless, our account of what community nurses say they do is an important step towards understanding how their experiences may contribute to current challenges in recruiting and retaining them. This understanding is essential if those challenges are to be addressed.

## 6 Implications and Recommendations

Our findings should encourage policy‐makers to continue supporting and developing an extension of community nurses' roles in palliative and end‐of‐life care. However, it should also remind them that this extension is currently associated with a level of unhappiness that is likely to threaten recruitment and retention. To mitigate this threat, we suggest two changes based on the findings of this study, in addition to recommendations about improving pay and involving nurses in any future plans to change their roles.

First, workforce planning needs to factor in enough capacity to enable nurses to engage in supported experiential learning about palliative and end‐of‐life care, both as learners and facilitators. Alongside this expansion of capacity, we recommend that nurse managers should embed regular clinical supervision within their provision of continuing education, giving it equal prominence to training courses.

Second, community healthcare systems need to provide a consistent level of medical back‐up that nurses are comfortable with; future research and evaluation should clarify the best ways of achieving this within a variety of health systems. Both these suggestions sit within a broader programme of organisational change that aims to support and strengthen collaborative relationships between healthcare professionals, prioritising and investing in their development as reflective practitioners.

## 7 Conclusion

We have presented a thick description of community nurses' experiences of providing palliative and end‐of‐life care in recent years. Key findings included the prevalence of positive responses to taking on extended responsibilities, the moral distress related to training and mentoring junior colleagues as well as to patient care and the widespread dissatisfaction with GP back‐up.

## Author Contributions

All authors have agreed on the final version. Each contributed to the following aspects of the study: B.B. and K.P.: conception and design, funding acquisition, data collection and interpretation and critical revision of the article. S.E.: conception and design, data collection and critical revision of the article. A.L. and S.B.: conception and design, funding acquisition and critical revision of the article. L.P.: data collection and interpretation, drafting and critical revision of the article.

## Disclosure

The views expressed are those of the authors and not necessarily those of the NIHR or the Department of Health and Social Care.

## Conflicts of Interest

The authors declare no conflicts of interest.

## Supporting information


Appendix S1

Appendix S2

Appendix S3

## Data Availability

The anonymised quantitative data used in this study may be requested by researchers through contacting the corresponding author. Given the sensitive subject nature of the anonymised qualitative data, it may be made available to researchers with evidence of a study protocol and Research Ethics Committee approval, and on completion of a data use and sharing agreement.
