# Supplementary material for: ‘We've Taken on a More Advanced Clinical Role’: A Multimethod Study of Community Nurses' Extended Roles in Palliative Care
Source: J Adv Nurs. 2025 Jun 17;82(2):1681–91. doi: 10.1111/jan.70019 (PMC12810649; doi:10.1111/jan.70019)
Supplement: Supplementary file 1 — Appendix S1 [file JAN-82-1681-s001.docx]

To describe participants’ level of seniority we refer to their “band”: banding is a pay structure and grading system used in the UK's National Health Service (NHS) to categorise nurses based on their responsibilities and the skills required for the role. “Band 5” nurses are typically newly-qualified; higher bands indicate higher pay and increasing levels of responsibility.

**E-survey participants characteristics**

Fifty-one participants took part in the e-survey: 18 (36%) were working as band 5 nurses, 14 (28%) as band 6 nurses, and 19 (37%) as band 7 or higher nurses. The majority of participants (n=30, 59%) worked in mixed urban and rural settings, while 8 (16%) reported working only in inner city areas and 8 (16%) in urban areas; five participants (10%) worked only in rural areas. Ten (20%) had up to five years’ experience working in the community, 28 (55%) reported they had been 6-15 years community experience, and 13 (25%) had been working for more than 15 years in the community. Respondents were from across the UK, although many (n=17, 33%) worked in the Southeast England and East of England (n=9, 18%).


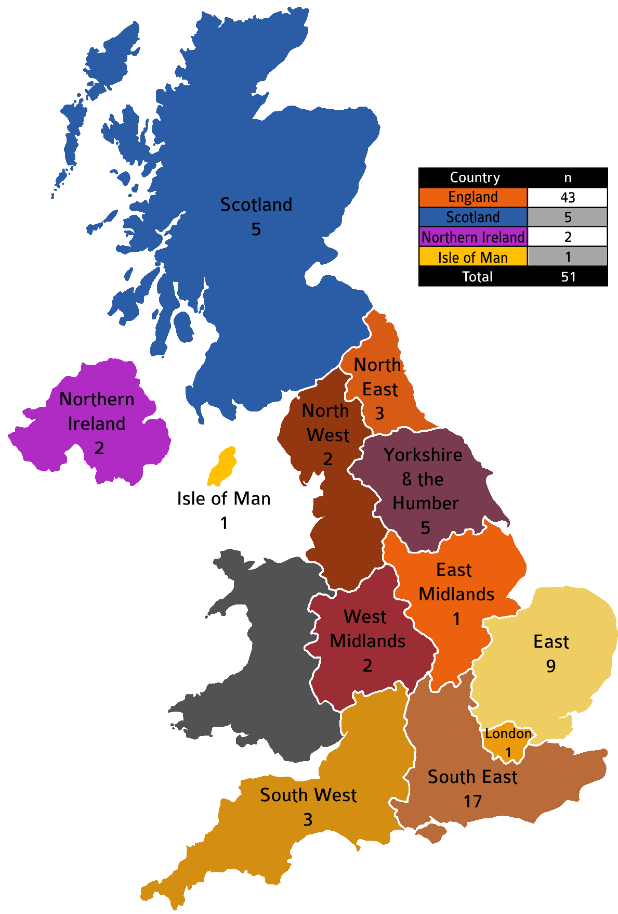


**Figure 1.** Distribution of e-survey respondents across the United Kingdom

**Focus group participants**

Thirty-five participants also took part in focus groups. Six (17%) had up to five years’ experience working in the community, 19 (54%) reported they had been working in the community for 6-15 years, and 10 (29%) had been working in the community for more than 15 years.

| **Focus Group Number** | **Number of participants** | **Bands of participants** |
| --- | --- | --- |
| One | Seven | Five band 6 and two band 7 nurses |
| Two | Two | Two band 6 nurses |
| Three | Four | Four band 6 nurses |
| Four | Seven | Seven band 5 nurses |
| Five | Two | One band 6 and one band 8a nurse |
| Six | Four | Four band 7 nurses |
| Seven | Three | One band 6 and two band 7 nurses |
| Eight | Two | Two band 7 nurses |
| Nine | Two | Two band 5 nurses |
| Ten | Two | Two band 5 nurses |
