# Supplementary material for: ‘We've Taken on a More Advanced Clinical Role’: A Multimethod Study of Community Nurses' Extended Roles in Palliative Care
Source: J Adv Nurs. 2025 Jun 17;82(2):1681–91. doi: 10.1111/jan.70019 (PMC12810649; doi:10.1111/jan.70019)
Supplement: Supplementary file 2 — Appendix S2 [file JAN-82-1681-s003.pdf]

UNIVERSITY OF CAMBRIDGE

PELiCam Palliative & End of Life Care in Cambridge

Participant e-survey text

Name of study: Community nurses' experiences of taking on extended clinical roles in end-of-life care during Covid-19. **Study ID number:** *[research team to complete]* _ _ _ _ _ _ _

What is your current professional role? Please tick the box that best describes your role

**Community staff nurse**		**District nurse** (team leader / specialist practitioner qualification)	
**Advanced nurse practitioner**		**Nurse consultant**	
**Community Matron**		**Other (please specify)**	
**Palliative care nurse specialist**			
**Nursing Home Nurse**			

What is your current NHS clinical band (or equivalent)? Please tick one

**Band 5**	
**Band 6**	
**Band 7**	
**Band 8a**	
**Band 8b and above**	

Which part of the United Kingdom do you work in? Please tick the main one

**England**	
**Scotland**	
**Wales**	
**Northern Ireland**	
**Other area (please specify)**	

Which county do you work in? Please state the main county

What type of area do you mainly work in? Please tick the main one

**Inner-city**	
**Urban**	
**Mixed urban and rural**	
**Rural**	
**Very rural / remote communities**	

UNIVERSITY OF CAMBRIDGE

PELiCam Palliative & End of Life Care in Cambridge

How many clinical staff are there in the community nursing team you work in? Please provide the total number. This includes trained nurses and healthcare support workers

Is your community nursing team based in the same building as the general practitioners you work with?

Yes	
No	

How many years of experience do you have of caring for people in the community? This may include time spent in other roles prior to your current role

0 - 2 years	
3 - 5 years	
6 - 10 years	
11 - 15 years	
16 - 20 years	
21 years or more	

How often do you personally care for terminally ill people in their last year of life in the community?

Every working day	
Several times each week	
Once a week	
A few times each month	
Once a month	
A few times each year	
Every few years	
Other (please specify)	

Please state whether your role in the following areas of end-of-life care has changed since the onset of the Covid-19 pandemic. Please circle one answer for each of the following statements

Having advance care planning conversations with patients and families

1<br>Doing a lot less	2<br>Doing less	3<br>Stayed the same	4<br>Doing more	5<br>Doing a lot more

UNIVERSITY OF CAMBRIDGE

PELiCam Palliative & End of Life Care in Cambridge

Asking patients and families about their preferences regarding cardiopulmonary resuscitation

1<br>Doing a lot less	2<br>Doing less	3<br>Stayed the same	4<br>Doing more	5<br>Doing a lot more

Completing the ReSPECT form / do not attempt cardiopulmonary resuscitation (DNACPR) documentation

1<br>Doing a lot less	2<br>Doing less	3<br>Stayed the same	4<br>Doing more	5<br>Doing a lot more

Making clinical decisions about the prescribing of anticipatory medications (injectable medications)

1<br>Doing a lot less	2<br>Doing less	3<br>Stayed the same	4<br>Doing more	5<br>Doing a lot more

Prescribing anticipatory medications, yourself

1<br>Doing a lot less	2<br>Doing less	3<br>Stayed the same	4<br>Doing more	5<br>Doing a lot more	N/A<br>Not a prescriber

Initiating / requesting the remote prescribing of anticipatory medications

1<br>Doing a lot less	2<br>Doing less	3<br>Stayed the same	4<br>Doing more	5<br>Doing a lot more

Using remote consultations (phone and video) with palliative patients and their families

1<br>Doing a lot less	2<br>Doing less	3<br>Stayed the same	4<br>Doing more	5<br>Doing a lot more

Providing in-person support visits for patients at the end of life and their families

1<br>Doing a lot less	2<br>Doing less	3<br>Stayed the same	4<br>Doing more	5<br>Doing a lot more

UNIVERSITY OF CAMBRIDGE

PELiCam Palliative & End of Life Care in Cambridge

Carrying out joint virtual consultations with general practitioners: where you are with the patient and family and the general practitioner joins you by phone or video

1<br>Doing a lot less	2<br>Doing less	3<br>Stayed the same	4<br>Doing more	5<br>Doing a lot more

Collaborative working with general practitioners

1<br>Doing a lot less	2<br>Doing less	3<br>Stayed the same	4<br>Doing more	5<br>Doing a lot more

Collaborative working with specialist palliative care teams

1<br>Doing a lot less	2<br>Doing less	3<br>Stayed the same	4<br>Doing more	5<br>Doing a lot more

Meeting with the multidisciplinary team, in-person or remotely, to discuss patients identified as being in their last year of life. For example, through Gold Standards Framework meetings.

1<br>Doing a lot less	2<br>Doing less	3<br>Stayed the same	4<br>Doing more	5<br>Doing a lot more

Making clinical decisions about end-of-life symptom management

1<br>Doing a lot less	2<br>Doing less	3<br>Stayed the same	4<br>Doing more	5<br>Doing a lot more

Administering (giving) injectable medications to manage symptoms in the last days of life

1<br>Doing a lot less	2<br>Doing less	3<br>Stayed the same	4<br>Doing more	5<br>Doing a lot more

Verifying deaths

1<br>Doing a lot less	2<br>Doing less	3<br>Stayed the same	4<br>Doing more	5<br>Doing a lot more	N/A<br>Not an option in my area

Providing bereavement support for family members and carers

1<br>Doing a lot less	2<br>Doing less	3<br>Stayed the same	4<br>Doing more	5<br>Doing a lot more

Thank you for completing this survey
