# Supplementary material for: ‘We've Taken on a More Advanced Clinical Role’: A Multimethod Study of Community Nurses' Extended Roles in Palliative Care
Source: J Adv Nurs. 2025 Jun 17;82(2):1681–91. doi: 10.1111/jan.70019 (PMC12810649; doi:10.1111/jan.70019)
Supplement: Supplementary file 3 — Appendix S3 [file JAN-82-1681-s002.pdf]

Semi-structured topic guide

Name of study: Community nurses' experiences of taking on extended clinical roles in end-of-life care during Covid-19

Note: Questions will be continually reshaped in response to data from previous focus groups and interesting facets raised by participants

Instructions for focus group facilitator

- The focus group is 60-minutes in length.
- **Please go through the 'introduction'** ***at the start of the session***, which will take approximately 5 minutes. ***All the ground-rules must be highlighted, especially confidentiality and the need to maintain patient/colleague anonymity.***
- The focus group covers three topics. It is not essential to cover every prompt under each topic - these are there to help direct and focus the discussion, but you are free to use your own discretion.
- **Try to let the conversation flow between group members**, rather than interrupting with prompts and probes. Use prompts if the group is quiet or it is necessary to refocus discussion.
- **After the focus group, please write a short (2-3 paragraph) reflection about how you felt it went as soon as possible afterwards.** It would be helpful to have your comments on group dynamics or the tone/mood of the session, as well as how you felt as the facilitator. There is a blank page at the end of the topic guide where you can record your reflections.
- **Please record the online focus group using the record function on Zoom or Teams.**

Facilitator's introduction (5 minutes)

1) **The facilitator introduces self**

2) **The facilitator briefly explains:** the purpose of the discussion is to understand community nurses' views and experiences of taking on extended clinical roles and activities in end-of-life care since the start of the pandemic. **An extended clinical role is any working practices that were not part of your usual work pre-pandemic. This might include making more complex clinical decisions, being more involved in prescribing decisions, having advance care planning conversations with patients and families that other professionals may have had previously.** We are interested in finding out as much as possible about these changes and the extended roles that you took on – what worked well, what did not work so well, and how things might be improved in the future.

3) **The facilitator explains the ground rules for the focus group discussion:**
   - We want you to do the talking, one person at a time
   - We would ideally like to hear from everyone, but you are free to participate as much or as little as you like
   - There are no right or wrong answers: please be sensitive to everyone's views and experiences.
   - What is shared in this room stays in this room: please keep everything you hear confidential When discussing examples from your own practice and teams, please refer to individuals by their role rather than names, or any identifiable details.

Focus group discussion (55 minutes):

Introduction (5 minutes):

Ask participants to introduce themselves by giving their name, job title and how long they have been involved in supporting people at the end of life in the community.

Topic 1. How have things changed (15 minutes):

1) **How have things changed for you, and those you work, with in providing end-of-life care since the onset of the pandemic?**

- *Ask for examples to illustrate points made – could you explain a little more about what that has looked like in practice?*

2) Some doctors and nurses reduced their face-to-face patient contacts during the pandemic, using home or video contacts instead. How has this affected your own work with patients approaching the end of life?

- *Ask for examples to illustrate points made – could you explain a little more about what that has looked like in practice?*

Topic 2. Effects of taking on extended clinical roles (15 minutes):

3) How do you feel changes in your clinical roles / working practices have influenced patient and family care at the end of life?

- *Ask for examples to illustrate points made – could you explain a little more about what has helped patients' and families experiences, what has not helped, and why?*
- ***Explore both positive and negative impacts***

4) How do you feel changes in your clinical roles regarding end-of-life care have affected the way you work with colleagues, including general practitioner and specialists?

- *Ask for examples to illustrate points made – could you explain a little more about why this has helped or been a hinderance?* ***Explore positive and negative effects.***
- *Have more remote ways working and communicating with colleagues had an influence?*

Topic 3. Development opportunities (15 minutes):

5) Looking to the future, which changes in ways of practicing would you like to keep?

6) Are there some changes you think should be reversed?

7) Do you feel you and your community nursing team have the training and resources needed to take on extended clinical roles in end-of-life care? If not, what additional resources and training is needed to make this sustainable?

- *Prompt. Who is best placed to support improvements / the consolidation of skills?*

Close (5 minutes):

- **Go around the group asking if they have other comments they wish to make**
- **Thank group for participating. Re-iterate confidentiality**

Facilitator reflections

Please write a short (2-3 paragraph) reflection on the next page about how you felt the session went as soon as possible afterwards and certainly on the same day. Reflect on group dynamics or the tone/mood of the session, were there any surprising responses from participants, as well as how you felt as the facilitator.

Facilitator reflection:

Facilitator name:

Date:

Focus group ID number:

Number of participants:

Facilitator reflective notes (continue on next page):
